# Spark Plasma Sintering of Commercial Zirconium Carbide Powders: Densification Behavior and Mechanical Properties

**DOI:** 10.3390/ma8095289

**Published:** 2015-09-10

**Authors:** Xialu Wei, Christina Back, Oleg Izhvanov, Oleg L. Khasanov, Christopher D. Haines, Eugene A. Olevsky

**Affiliations:** 1 Department of Mechanical Engineering, College of Engineering, San Diego State University, 5500 Campanile Dr., San Diego, CA 92182, USA; E-Mail: xwei@mail.sdsu.edu; 2 General Atomics, 3550 General Atomics Ct., San Diego, CA 92121, USA; E-Mails: tina.back@ga.com (C.B.); oleg.izhvanov@ga.com (O.I.); 3 National Research Tomsk Polytechnic University, Tomsk 634650, Russian; E-Mail: khasanov@tpu.ru; 4 US Army Armament Research Development Engineering Center, Picatinny Arsenal, NJ 07806, USA; E-Mail: christopher.d.haines2.civ@mail.mil

**Keywords:** zirconium carbide (ZrC), spark plasma sintering (SPS), power-law creep (PLC), transverse rupture strength (TRS), microhardness (*H*_v_)

## Abstract

Commercial zirconium carbide (ZrC) powder is consolidated by Spark Plasma Sintering (SPS). Processing temperatures range from 1650 to 2100 °C. Specimens with various density levels are obtained when performing single-die SPS at different temperatures. Besides the single-die tooling setup, a double-die tooling setup is employed to largely increase the actual applied pressure to achieve higher densification in a shorter processing time. In order to describe the densification mechanism of ZrC powder under SPS conditions, a power-law creep constitutive equation is utilized, whose coefficients are determined by the inverse regression of the obtained experimental data. The densification of the selected ZrC powder is shown to be likely associated with grain boundary sliding and dislocation glide controlled creep. Transverse rupture strength and microhardness of sintered specimens are measured to be up to 380 MPa and 24 GPa, respectively. Mechanical properties are correlated with specimens’ average grain size and relative density to elucidate the co-factor dependencies.

## 1. Introduction

As a typical ultra-high temperature ceramic, zirconium carbide (ZrC) offers good thermo-mechanical properties, high electrical and thermal conductivity, high melting temperature and strong chemical resistance. It has recently been considered to be a promising candidate for high temperature applications, such as furnace elements, arc plasma electrodes and future nuclear reactors [1,2,3,4]. ZrC also possesses a high melting point of 3532 °C, which, together with the inherent nature of the covalent character of Zr-C bonding, leads to the ineffective sintering of ZrC powders, if conducted at relatively low temperatures. Therefore hot powder consolidating techniques have to be commonly employed to prepare bulk ZrC products, most of time high processing temperatures [5] and large external pressures are required to be applied simultaneously [6].

Hot-pressing (HP) was first used to consolidate ZrC powder based materials [7,8,9,10]. With the assistance of uniaxial pressure, ZrC bodies with high relative densities (≥98%) were obtained at 2300 °C by Barnier *et al.*, via hot pressing [7]. In order to improve the sinter-ability of ZrC powders, metallic and oxide sintering additives were used. A similar relative density level was reached at relatively lower temperatures (1900–2000 °C) by adding zirconium [8] and yttria-stabilized zirconia [9] in hot pressing of ZrC powders. However, hot pressing technique always accompanies large grain growth [10] which compromises the mechanical properties of the final product. In addition, the presence of the second phase (sintering aids) may limit its applications in some extreme situations. High density ZrC body with fine microstructure is therefore needed to be produced from ZrC powders without sintering aids via advanced powder consolidation techniques.

Spark plasma sintering (SPS), also known as filed-assisted sintering technique (FAST) or current-assisted sintering technique, has been evidenced as a promising way to densify hard and refractory materials. During SPS processing, the electric current goes through the tooling and the sample to heat them up rapidly through Joule heating. Therefore the target processing temperature can be reached shortly. Also, in some cases, the applied pressure largely offsets the temperature effects. Therefore SPS can actually consolidate powder components at lower temperature while preventing unnecessary grain growth. Various benefits of applying field-assisted sintering techniques have been elaborated in a number of review papers [11,12,13,14].

SPS has been applied to consolidate ZrC powders since 2006. Ryu *et al.*, first reported the 63% relative density reached at 1500 °C under 30 MPa, while the addition of Dy_2_O_3_ promoted the increase of the relative density level by 5% [2]. Undoped ZrC was densified by Sciti *et al.*, at 2100 °C under 65 MPa [15]. The mechanical properties of the final product, such as hardness and elastic modulus were also included in their study. Gendre *et al.* synthesized the submicron sized zirconium oxy-carbide (ZrC*_x_*O*_y_*) powders using the carboreduction of zirconia powder and fully sintered them around 2000 °C [1]. The authors also stated that the existence of oxygen and vacancies could enhance the densification kinetics of synthesized ZrC*_x_*O*_y_* powder in another study [4]. High energy ball milling was employed to bring the crystallite size of as-purchased ZrC powder down to nano range, in such a way that the densification kinetics were significantly improved and a finer microstructure was then obtained [16]. Nano-scaled ZrC powders were also prepared from a novel sol-gel method by Xie *et al.*, recently [17]. Up to 99% relative density was achieved and limited grain growth was observed as well after processing the obtained powders via SPS at 1750 °C.

The densification mechanism under SPS processing also drew researchers’ attention. Research interests included the role of electrical current and field effects [18,19,20,21], high heating rate [22], diffusional effects [23,24], and non-linear (power-law) creep under high temperature and pressure [1,25,26]. In order to elucidate the creep mechanism of ZrC powder undergoing SPS, Gendre *et al.* estimated the creep exponent, *n*, and the activation energy, *Q*, of ZrC undergoing the dwelling stage of SPS by using an empirical model [1] based on Ashby’s hot pressing diagrams [27].

Li *et al.*, developed the so-called multi-step pressure dilatometry (MSPD) approach to investigate the stain rate sensitivity exponent, *m*, of copper powder subjected to SPS. This novel approach bypasses the influence of microstructure evolution by suddenly increasing the applied pressure to obtain short term stress-strain relation [26].

In the present work, instead of utilizing submicron or nano-sized ZrC powders from a special synthesis procedure or ball-milling as in the above-mentioned publications, a commercial micron-sized ZrC powder is used directly. To effectively densify the selected powder, SPS runs are performed at different temperatures. Both single-die and double-die SPS tooling setups are hired to obtain the densification kinetics of the employed powder. The experimental results are compared to those of the previously conducted studies. The densification mechanism of this ZrC powder under SPS conditions is investigated utilizing a power-law creep constitutive equation, whose coefficients are determined by the inverse regression of the obtained experimental data. The average grain size and relative density of the sintered specimens are evaluated and correlated with the specimen’s mechanical properties, such as transverse rupture strength and microhardness, to determine their co-effect on the aimed properties.

## 2. Mathematical Model of Power-Law Creep of Powder Materials

Power-law creep has been considered to be the dominant mechanism to describe the viscoelastic behavior of crystalline solids [28]. A widely accepted model of power-law creep proposed by previous studies can be expressed as [7,29]:

(1) $$\dot{\varepsilon }=\frac{A}{T}\;exp\left(-\frac{Q}{RT}\right)\sigma^{n}$$

where *A* is a combined material constant; *T* is the specimen’s temperature; *Q* is the activation energy, *R* is the gas constant; *n* is the stress exponent; and $\dot{\text{ε}}$ is the strain rate. Based on the format of Equation (1), by considering a porous body subjected to an externally applied pressure, Olevsky derived a constitutive equation to explicitly describe the stress-strain states during sintering of porous materials in the form [30]:

(2) $$\sigma_{ij}=A_{cr}W^{m-1}\left[\varphi {\dot{\varepsilon }}_{ij}+\left(\psi -\frac{1}{3}\varphi \right)\dot{e}\delta_{ij}\right]+P_{L}\delta_{ij}$$

where $\sigma_{ij}$ is the external pressure; $A_{cr}$ is the creep coefficient (see below) [31]; $A_{m}$ is also a material parameter.

(3) $$A_{cr}=A_{m}T^{m}exp\left(\frac{mQ}{RT}\right)$$

*m* is the strain rate sensitivity exponent, m=1/n. *W* is the equivalent effective stain rate,

(4) $$W=\frac{1}{\sqrt{1-\theta }}\;\sqrt{\varphi {\dot{\gamma }}^{2}+\psi {\dot{e}}^{2}}$$

With $\dot{\text{γ}}$ corresponding to the shape change rate; $\dot{e}$ represents the volume change rate. Parameters φ and ψ are the normalized shear and bulk modulus, respectively. Both of them can be written in terms of porosity or the void fraction of the processed material, θ, as [32]:

(5) $$\varphi ={\left(1-\theta \right)}^{2}$$

(6) $$\psi =\frac{2}{3}\frac{{\left(1-\theta \right)}^{3}}{\theta }$$

$P_{L}$ is the effective sintering stress; in the case of a pressure-assisted sintering (e.g., SPS or hot pressing), it is often negligible. Parameter $\delta_{ij}$ is Kronecker’s delta ($\delta_{ij}=0\;\text{if}\;i=j,\;\delta_{ij}=0\;\mathrm{if}\;i\neq j)$. In this study, a cylindrical coordinate system is considered. Taking into account the boundary conditions, the radial strain rate, ${\dot{\varepsilon }}_{r}$, is equal to zero in a system which is constrained along the radial direction. The shape change rate, $\dot{\gamma }$, and the volume change rate, $\dot{e}$, are thereby expressed by the axial strain rate, ${\dot{\varepsilon }}_{z}$, as [26]:

(7) $$\dot{\gamma }=\sqrt{\frac{2}{3}}\left|{\dot{\varepsilon }}_{z}-{\dot{\varepsilon }}_{r}\right|=\sqrt{\frac{2}{3}}\left|{\dot{\varepsilon }}_{z}\right|$$

(8) $$\dot{e}={\dot{\varepsilon }}_{z}+2{\dot{\varepsilon }}_{r}={\dot{\varepsilon }}_{z}$$

According to mass conservation, the volume change rate can be also calculated through the evolution of the specimen’s porosity as shown below:

(9) $$\frac{\dot{\theta }}{1-\theta }=\dot{e}$$

Equations (3)–(9) can be substituted into the formulated Equation (2) to obtain:

(10) $$\dot{\theta }=\frac{d\theta }{dt}=-{\left(\frac{\sigma_{z}}{A_{m}T^{m}exp\left(\frac{mQ}{RT}\right)}\right)}^{\frac{1}{m}}{\left(\frac{3\theta }{2}\right)}^{\frac{m+1}{2m}}{\left(1-\theta \right)}^{\frac{m-3}{2m}}$$

where $\sigma_{z}$ is the applied axial stress. Equation (10) describes how porosity changes with time at given sintering temperatures and pressures. The densification mechanism can be revealed through the determination of the power-law creep exponent *m* (see Section 4.2.).

## 3. Experimental Procedures

### 3.1. Starting Powders

**Figure 1:**
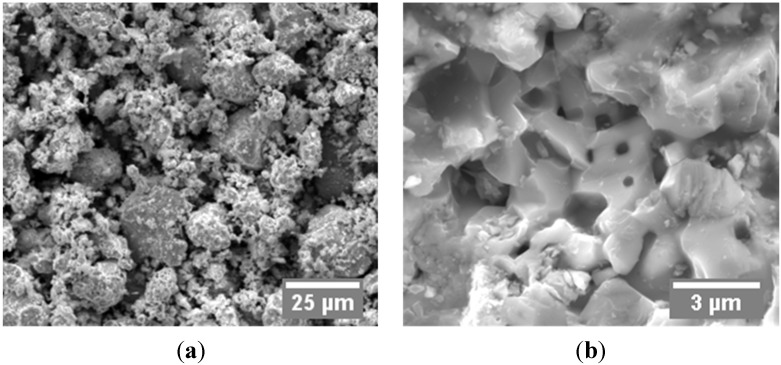
SEM images of as received ZrC powder: (**a**) 1000×; (**b**) 10000×.

A commercial zirconium carbide powder (ZrC, 99%, Sigma-Aldrich Co., St. Louis, MO, USA) was chosen as the tested material in the present study. The purity of the as received powder was higher than 99%. Scanning electron microscopy (SEM: Quanta 450, FEI Co., Hillsboro, OR, USA) was used to examine the morphology of the original powder, as shown in Figure 1. The received powder was a combination of small flakes and large agglomerates with an average particle size of 10 µm (Figure 1a). A single particle shows a polycrystalline structure and, when examining under high magnifications, inter- and intra-granular pores are observed as well (Figure 1b). The density of the initial particles was measured to be 6.498 g/cm^3^, by Helium absorption pycnometer (AccuPyc 1330, Micromeritics Instruments Corp., Norcross, GA, USA). Due to the existence of the internal particle porosity, the measured density is slightly lower than the computed theoretical density of ZrC based on the crystallographic properties (6.70 g/cm^3^ [33]). The energy dispersive X-ray spectroscopy (X-Max-50 detector, Oxford Instruments, Concord, MA, USA) in SEM mode determined the atomic ratio (at. %) between zirconium and carbon as close to 1. Above-discussed powder properties are listed in Table 1.

**Table 1:** Properties and compositions of as received ZrC powder.

Powder Supplier	Absolute Density (g/cm^3^)	Average Particle Size (µm)	Average Grain Size (µm)	Zr (at. %)	C (at. %)	O (at. %)
Sigma Aldrich	6.498	10	<1 µm	44.74	52.91	2.35

### 3.2. Spark Plasma Sintering Process

All the SPS consolidation runs of as received ZrC powder were carried out with Dr. Sinter SPSS-515 furnace (Fuji Electronic Industrial Co., Ltd., Kawasaki, Japan). This apparatus is equipped with a maximum axial uniaxial loading ability of 50 kN and an output DC current up to 1500 A. For each single-die SPS experiment, approximate 5 grams of ZrC powder were placed in a graphite die with an internal diameter of 15.3 mm and mounted between the upper and lower graphite punches with an external diameter of 15 mm (I-85 graphite, Electrodes Inc., Santa Fe Springs, CA, USA). Carbon paper with thickness of 0.15 mm (Fuji Electronic Industrial Co., Ltd., Kawasaki, Japan) was inserted between the powder bed and the graphite components in order to protect the die wall and ease the ejection of the specimen after sintering. The pre-compaction of the powder was conducted at room temperature by applying a minimum pressure (~3 kN) of the SPS apparatus to the ZrC powder through the graphite tooling located in the SPS device chamber with a holding time of 3 min. The geometrical dimensions of the green specimen at this point were used to calculate the initial relative density. Carbon felt was used to surround the graphite die with the purpose of reducing heat loss through thermal radiation.

The 12:2 on/off-interval pulsed current was chosen for all the experiments. The heating rate was 100 °C/min to 1600 °C, and then 50 °C/min to the peak temperature. A series of SPS tests were performed with peak temperatures ranging from 1650 to 1900 °C. Experimental runs at 2100 °C were also performed in order to compare with the published data and a constant 100 °C/min heating rate was used in that case. Temperature evolution was monitored by a digital radiation thermometer focused on the outer surface of the graphite die. When preparing specimens using the single-die SPS configuration [34], the axial pressure was kept at 60 MPa all the time. The specimens were held at the peak temperature for 25 min. Argon atmosphere was introduced into the SPS chamber when the peak temperature was higher than 1750 °C in order to prevent the chamber and electrodes from being overheated. The real-time processing parameters, such as temperature, applied force, and axial displacement, were automatically recorded by the SPS device. For every selected heating profile, an additional heating run was conducted in the absence of powder. The obtained axial readings from this blank run were considered to be the result of the thermal expansion of the graphite tooling under such a temperature regime. The true axial shrinkage of the specimen during consolidation was therefore possible to precisely evaluate.

**Figure 2:**
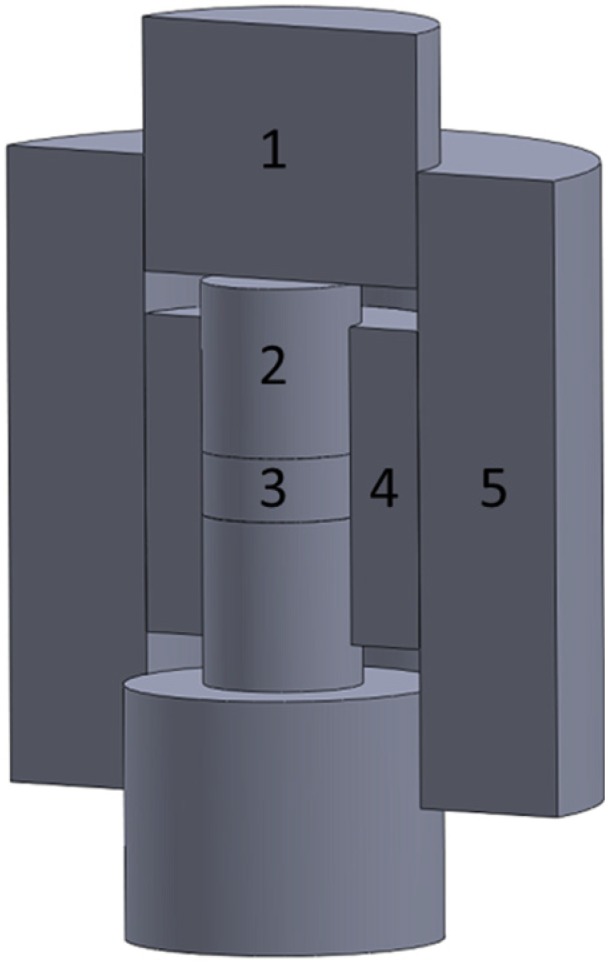
Schematics of the double-die SPS setup used in this study: 1—Large punch; 2—Small punch; 3—Specimen; 4—Small die; 5—Large die.

Due to the limited mechanical strength of the electrode graphite, at high temperatures, the regular 15.3 mm die cannot sustain the internal radial stress caused by the Poisson deformation of the powder bed under high axial pressure (>80 MPa). In order to build up high pressure, besides the regular single-die SPS tooling configuration, a double-die setup [35] was also utilized, as illustrated in Figure 2. A small graphite die with inner diameter of 7.3 mm and outer diameter of 15 mm was located at the center of the regular 15.3 mm graphite die. Two silicon carbide cylindrical punches with a diameter of 7 mm and a length of 7 mm (Morgan Technical Ceramics, Hudson, NH, USA) were used to hold the specimen within the small graphite die. Two 15 mm diameter graphite punches with length of 10 mm were subsequently aligned with the SiC punches to sustain and transmit the external force from the machine hydraulic system.

This double-die setup was used to investigate the densification and microstructure of ZrC powder specimen under high applied pressure. By applying the same level of force, the created pressure via double-die setup could be up to four times larger than that of the single-die setup as the specimen was subjected to a reduced loading area in the former case. Carbon papers were inserted between the specimen and the small die, and between the small and large graphite dies too. Double die SPS of ZrC was performed at 1800 °C under 200 MPa. Holding time was reduced to 10 min because of the increase of pressure.

### 3.3. Characterization of Processed Specimens

Every individual experiment was repeated at least twice, one specimen was used for the rupture test and the other one was prepared for characterizing cross-sectional properties, such as grain size and microhardness. The cooled specimens were ground to remove the adherent graphite paper from the outer surfaces. Their final densities were measured using the Archimedes method with deionized water. To ensure the accuracy, the specimens were coated with a thin layer of paraffin wax by a dip-coating method before applying the Archimedes measurements. In addition, by assuming a constant specimen radius during SPS, the true axial shrinkage was used to calculate the specimen’s instantaneous density change with respect to the processing time. The relative densities of the produced specimens were determined as the ratio of the specimen’s density to the theoretical density of ZrC (6.7 g/cm^3^). All specimens were well polished with the assistance of colloidal diamond suspension before performing any microstructural and mechanical analyses.

A particular transverse rupture strength test procedure was previously designed for specimens with a cylindrical shape [36]. By gradually increasing the applied force through a tungsten carbide ball-shaped indenter onto a disk-shaped specimen, the rupture strength of this specimen can be calculated as:

(11) $$\sigma_{TRS}=\frac{F}{h^{2}}\left[\left(1+\nu \right)\left(0.485log\frac{a}{h}+0.52\right)+0.48\right]$$

where *F* is the applied force upon the occurrence of rupture; *h* is the specimen thickness; ν is the Poisson’s ratio; and a is the effective specimen radius. The loading rate was set as 0.001 in/s (equivalent to 0.0254 mm/s). During the test, the applied force, *F*, was recorded by a materials testing device (5982, Instron Inc., Norwood, MA, USA). The Poisson’s ratio of partially dense material is determined by its porosity, θ, as [37]:

(12) $$\nu =2\nu_{0}\left(\frac{2-3\theta }{4-3\theta }\right)$$

where $\nu_{0}$ is the theoretical Poisson’s ratio of fully dense ZrC [38]. After the rupture test, large representative pieces of broken specimens were subjected to SEM to examine the microstructures of the fracture surface.

Another set of specimens were cut through the central plane by a high precision diamond saw. Both obtained specimen halves were hot mounted within Bakelite powder so that the two cross-sectional surfaces could be polished together using the automatic polishing machine. One half was placed on the microhardness testing machine (M-400-H1, Leco Corp., St. Joseph, MI, USA) to test the Vickers hardness (*H_v_*). This polished surface was indented by a standard diamond indenter at the center, at the midpoint and at the edge, all under a pre-set force of 0.5 kgf. The other half was examined using SEM to evaluate the grain size and microstructure. Before being microscopically observed, the polished surface was etched for 3 min using HF:HNO_3_:H_2_O solution in a volumetric ratio of 1:1:3 in order to have a better reflection of grain geometries. The average grain size was estimated by applying image analysis software, based on the mean linear intercept method with a correction factor of 1.5.

## 4. Results and Discussion

### 4.1. Densification Behavior and Microstructures

The initial and final relative densities of the SPS processed specimens are listed in Table 2. The geometrical-based final density from the true axial displacement is also included as a reference. As one can see from Table 2, the measured final densities from Archimedes method are comparable to those calculated from the geometrical method, which indicates that the both means of the measurement are reliable.

**Table 2:** Relative densities of sintered specimens.

Specimen #	Tooling Setup	Peak Temperature (°C)	Pressure (MPa)	Holding (min)	Initial Density	Final Density	
						Archimedes	Geometry
1	Single-die	2100	60	25	61.08%	97.28%	97.35%
2	Single-die	1900	60	25	61.16%	96.29%	96.37%
3	Single-die	1800	60	25	61.55%	95.16%	95.29%
4	Single-die	1750	60	25	61.65%	91.21%	91.18%
5	Single-die	1700	60	25	60.86%	81.52%	81.98%
6	Single-die	1650	60	25	60.08%	77.70%	77.31%
7	Double-die	1800	200	10	61.23%	97.87%	97.84%

The single-die SPS was successfully used to consolidate the employed ZrC powders to high density when temperature was above 1750 °C (Specimens # 1, 2, and 3). The specimens prepared at lower temperatures via single-die SPS (Specimens # 4, 5, and 6) were not able to reach high density. However, the relative density difference between specimens processed at 1900 °C and 2100 °C is not that obvious even despite of a temperature increase of 200 °C, which to some degree indicates the saturation of the temperature level importance for the densification intensity.

The effect of pressure on the densification can be seen when comparing the obtained final densities by the single-die SPS to those by the double-die processing case: the double-die SPS, under a pressure of 200 MPa, rendered the highest relative density even though it was conducted at 1800 °C.

**Figure 3:**
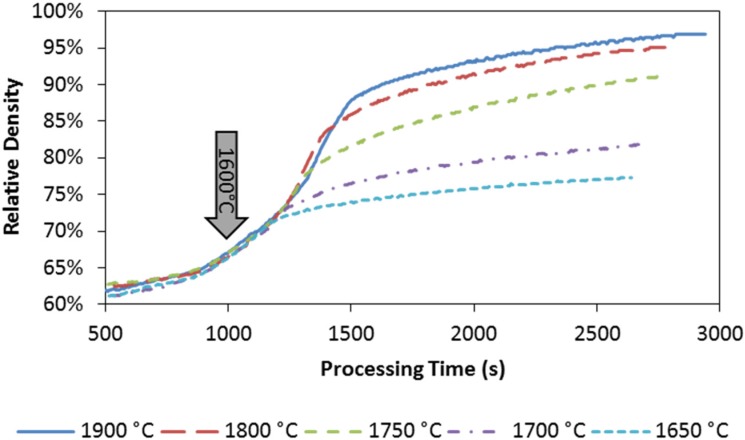
Relative density evolution with respect to processing time: single-die SPS at maximum processing temperature of 1900 °C, 1800 °C, 1750 °C, 1700 °C, and 1650 °C, respectively. Arrow is associated with the temperature of 1600 °C.

**Figure 4:**
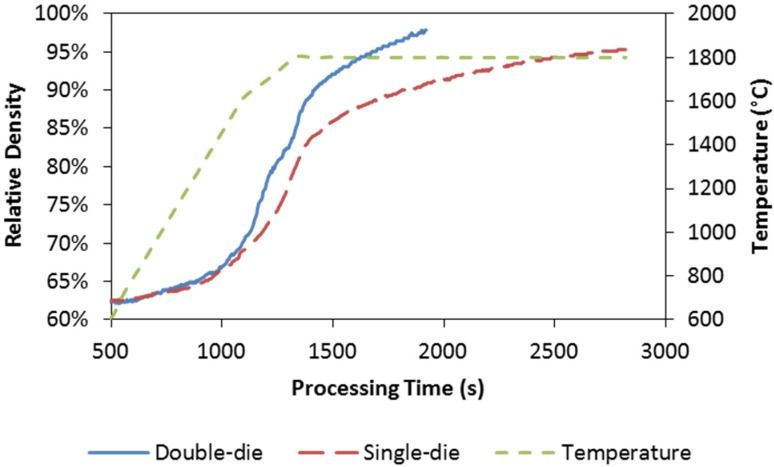
Relative density evolution with respect to processing time: double-die and single-die SPS at the maximum processing temperature of 1800 °C. Temperature evolution is included as secondary vertical axis.

The evolutions of the relative density with respect to processing time for the single-die and double-die SPS runs are plotted in Figure 3 and Figure 4.

It can be seen from Figure 3, that fast densification starts around 1000 s during the single-die SPS, which corresponds to a processing temperature around 1600 °C. Beyond this point, the specimen’s densification intensifies consistently as temperature ramps up to the target level. It is worth noticing that there is a threshold between 1700 and 1750 °C in terms of the densification rate (time derivative of the relative density). Faster densification appears above this threshold, while the slower rate is shown below it. The effect of temperature on the densification is obvious: the higher the maximum temperature is, the faster the specimen densifies. The densification kinetics of the double-die SPS at the maximum temperature of 1800 °C is compared to that of the single-die SPS in Figure 4. As the double-die setup enables much higher pressure to be applied to the specimen than the single-die setup, the densification kinetics is significantly improved. It also can be seen that the final density could go further if a longer holding time was allowed.

**Figure 5:**
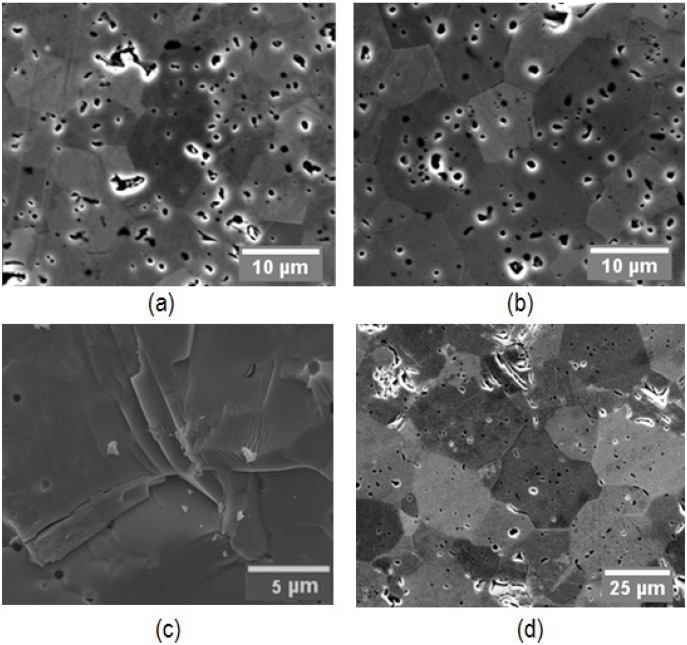
SEM images of fracture surfaces: (**a**) Single-die SPS at 1800 °C, Specimen #3; (**b**) Single-die SPS at 1900 °C, Specimen #2; (**c**) Single-die SPS at 2100 °C, Specimen #1; (**d**) Double-die SPS at 1800 °C, Specimen #7.

The microstructures of the polished cross-sectional surfaces were examined by the SEM (Quanta 450, FEI Co., Hillsboro, OR, USA). Micro-morphologies of the specimens prepared by the single-die SPS at maximum temperatures of 1800 °C, 1900 °C, and 2100 °C, as well as by double-die SPS at 1800 °C, are shown in Figure 5. The specimens prepared by the single-die SPS have similar microstructures except the level of the grain growth. Both inter- and intra-granular pores are present in the microstructures of these specimens. The amount and the size of the inter-granular pores are decreasing as relative density increases (Figure 5a–c), but some intra-granular pores are not showing the same level of shrinkage even in the specimens obtained at 2100 °C with nearly no inter-granular pores present (Figure 5c). Note that different scale bars are used in these SEM images.

Figure 5d shows a typical microstructure of the specimens produced by the double-die SPS, in which the amount and the size of intra-granular pores significantly drops down compared to the observed morphologies resulted from the single-die SPS.

It should be noted also that intra-granular pores are present in most specimens. The complete elimination of residual porosity, especially of the intra-granular one, requires further manipulation of temperature and loading regimes.

Also, the double-die SPS at 1800 °C results in comparable or even larger grain sizes than those obtained from single-die SPS at 2100 °C. The grain growth in double-die SPS can be explained as follows: in a double-die setup, on the one hand, the reduced contact area between the large and small punch can cause the occurrence of local overheating, which has been previously reported [39]. On the other hand, the insert of the small die and punches can introduce extra contacts to the system and contribute to an additional increase of the temperature [40]. In the double-die SPS, high pressure drives the densification and the closure of internal porosity but overheating appears to be more dominant to the grain growth phenomenon. Finer microstructures may be obtained by lowering the peak processing temperatures.

### 4.2. Densification Mechanism

Major mechanisms of power-law creep are defined by specifying the value of the exponent, *n* or *m*, (see Equation (1)) in the following categories,

- Diffusional creep, n=1 (m=1), models were developed by Herring [41] and Nabarro [42], also known as Nabarro-Herring creep.
- Grain boundary sliding based creep, n=2 (m=0.5), suggested by Gifkins in 1976 [43].
- Dislocation glide controlled creep, n=3 (m=0.33), proposed by Weertman when explaining prismatic glide creep under low stress conditions [44].
- Dislocation climb-controlled creep, n=3−5 (m=0.2−0.33), also reported by Weertman in early publications [45,46].

Thus, in the present study we employ a phenomenological approach for the determination of the mass transport mechanism driving densification during spark-plasma sintering. A significant advantage of this approach is in its capacity of using only an *in situ* macroscopic displacement data to clarify the ZrC deformational mechanisms and to directly obtain the values of the important constitutive parameters, such as the coefficients of power-law creep (strain rate sensitivity “*m*”, in particular). It should be noted that similar approaches have been utilized in the above-mentioned works of Herring, Nabarro, Gifkins, Weertman, *etc.* [41,42,43,44,45,46]. The aforesaid classic studies, however, have been focused on the determination of the creep properties of fully-dense materials, while the present work extrapolates this well-established methodology to the domain of porous (compressible) materials. The obtained values of the creep parameters can be directly used in the respective finite-element codes [31] developed for solving concrete SPS problems and enabling the predictions of the evolution of the relative density, stresses, strains, temperature, and electric current density spatial distributions.

It should be noted that for a more complete multi-scale analysis of mass transport mechanisms, the utilized phenomenological approach should be complemented by the direct nano- or atomic-scale observations of the mass transfer topological specifics (evidences of grain-boundary sliding or dislocation climb, *etc.*). These topological evidences are rather difficult to obtain. One of the first publications including a detailed analysis of the microstructure and pore evolution during SPS of TaC [47] provides an example of such a study. A combination of the macroscopic phenomenological approach and a high resolution microscopic analysis of the atomic-scale mass transport is an important direction of future research.

**Figure 6:**
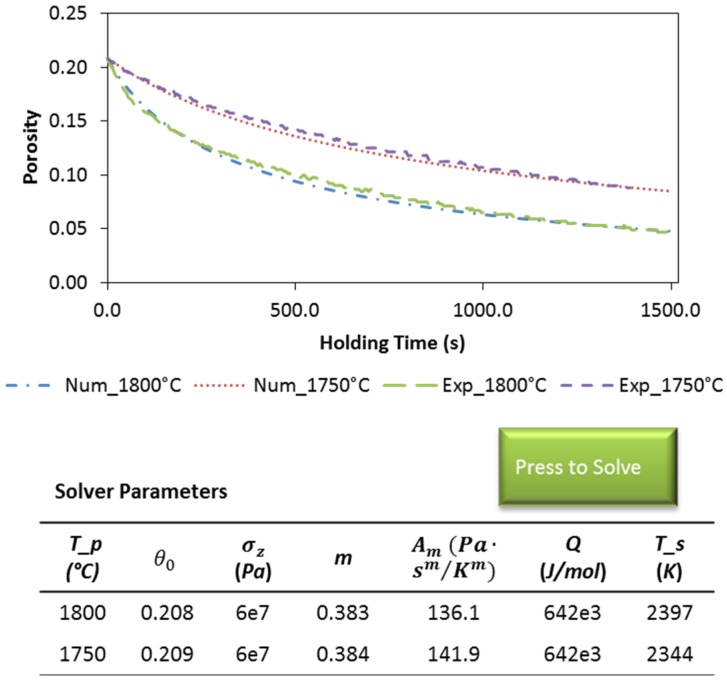
Numerical solver embedded spreadsheet interface: inverse regression of experimental data obtained from the holding stages in single-die SPS at 1800 °C and 1750 °C, respectively.

The axial pressure, $\sigma_{z}$, is a known parameter in the conducted SPS experiments, and the specimen’s temperature, *T*, can be determined by using the previously developed finite element modeling framework [39]. The coefficients $A_{m},\;Q$ and m are necessary to be pre-determined in order to solve Equation (10) numerically. An inverse regression can be conducted to minimize the discrepancies between numerical and experimental curves by substituting different series of $A_{m},\;Q$ and m parameters till their optimal combination is found. To facilitate this “trial and error” procedure, the isothermal holding periods of the conducted SPS runs were selected as the representative stages, since the use of constant temperature values can reduce the target equation’s degrees of freedom. The porosity-time profiles of ZrC dwelled at maximum processing temperature of 1800 °C and 1750 °C in the single-die SPS were first considered. The numerical solver interface is presented in Figure 6. This way, Equation (10) can be solved numerically and plotted automatically to be compared with the experimental data. Choosing the proper values of $A_{m},\;Q$ and m provides a good agreement between the numerical and experimental curves. The numerical solution also indicates the role of temperature in the densification: when starting from the same porosity, each individual curve approaches its target density at a distinguishing rate.

In the same manner, the creep coefficients for the single-die SPS at 1900 °C and 2100 °C as well as the double-die SPS at 1800 °C were evaluated; the obtained power-law creep coefficients are listed in Table 3. All the obtained *m* values, although the one for the double die SPS being slightly higher than the others, fall in the range from 0.33 to 0.5 (equivalent to *n* varying from 2 to 3). The densification mechanism of the selected ZrC powder in the investigated temperature range is then shown to be likely associated with grain boundary sliding (*n* = 2) and dislocation glide controlled creep (*n* = 3). High levels of the apparent activation energy are exhibited, indicating the poor sinter-ability of the studied ZrC powder. Also, the activation energy appears with nearly no fluctuation no matter what processing temperature or applied load is used. The obtained results are close to the ones obtained in the study conducted by Gendre *et al* using finer powders from carboreduction of zirconia particles [1].

**Table 3:** Creep coefficients obtained from inverse regression of experimental data.

Specimen #	Tooling Setup	Temperature (°C)	Pressure (MPa)	*m*	*n*	*Q* (kJ/mol)
1	Single-die	2100	40	0.379	2.63	644
2	Single-die	1900	60	0.381	2.62	640
3	Single-die	1800	60	0.383	2.61	642
4	Single-die	1750	60	0.384	2.60	642
7	Double-die	1800	200	0.412	2.43	643

### 4.3. Transverse Rupture Strength of the Specimens Processed by SPS

Transverse rupture tests provide the specimens’ mechanical properties at a macro level. Test results are summarized in Table 4 together with respective average grain sizes. Double-die SPS specimens were not subjected to TRS test as they were too small to be mounted on the testing fixture. Maximum rupture strength was found to be around 380 MPa for the highest density specimens prepared at the maximum temperature of 2100 °C. This corresponds to the same level of results using three-point bending tests [15] and four-point bending tests [9] on rectangular shape ZrC specimens, which evidences the reliability of this simplified testing method for cylindrical shape specimens.

**Table 4:** Transverse rupture strength and microhardness test results.

Specimen #	Tooling Setup	Temperature (°C)	Relative Density	Ave. Grain Size (µm)	TRS (MPa)	H_V_ (GPa)
1	Single-die	2100	97.28%	30.92	378.8	23.66
2	Single-die	1900	96.29%	14.60	319.8	22.87
3	Single-die	1800	95.16%	11.42	270.1	21.29
4	Single-die	1750	91.21%	7.34	214.6	18.01
5	Single-die	1700	81.52%	5.12	154.8	11.56
6	Single-die	1650	77.70%	2.58	150.3	7.90
7	Double-die	1800	97.87%	37.08	N.A.	24.68

**Figure 7:**
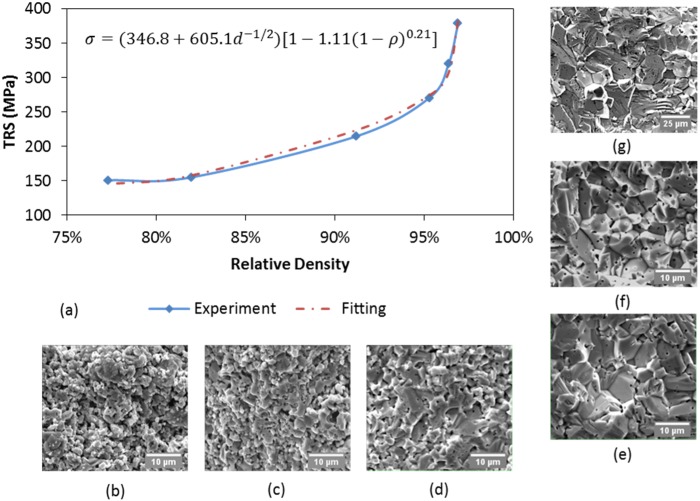
Transverse rupture test results and SEM images of fracture surfaces: (**a**) TRS results with respect to relative density; (**b**–**g**) SEM images of fractural surfaces of specimens sintered at 1650 °C, 1700 °C, 1750 °C, 1800 °C, 1900 °C, and 2100 °C, respectively. Fitting equation with optimized coefficients is also presented.

Test results are plotted with respect to the relative densities in Figure 7. The rupture strength largely depends on the relative density. Higher strength is associated with higher density. Sharp increase of the rupture strength is observed when the relative density goes over 95%. SEM images of fracture surfaces (see also Figure 7b–g) illustrate how the cross-sectional morphology changes with the relative density. A consolidated structure with clear grain boundaries starts appearing in the specimens obtained at 1750 °C (Figure 7d) and keeps growing in the specimens sintered at higher temperatures. As the processing temperature determines the level of densification as well as the grain growth, there is a great possibility that the sintering mass transfer mechanism transits from surface diffusion to grain boundary diffusion around 1750 °C, in agreement with what has been assumed in last section. These fracture surface images also suggest the fracture mechanism belongs to the category of inter-granular fracture.

Besides the relative density, the average grain size also needs to be taken into account as a factor that affects the rupture strength. The yield stress of a specimen can be related to its average grain size using Hall-Petch relationship [48]. After combining Hall-Petch relationship with the strength—porosity dependence of a porous body [49], an expression of transverse rupture strength (MPa), $\sigma_{TRS}$, can be obtained taking into account the influence of the average grain size (µm), *d*, and the relative density, ρ.

(13) $$\sigma_{TRS}=(k_{1}+k_{2}d^{-1/2})\left[1-k_{3}{\left(1-\rho \right)}^{a}\right]$$

where $k_{1},\;k_{2},\;k_{3},$ and a are parameters used to fit the experimental data. By substituting the average grain size and relative density from Table 4 into Equation (13), fitting parameters are optimized to make a good reproduction of the experimental data, as illustrated in Figure 7a.

### 4.4. Microhardness of the Specimens Processed by SPS

**Figure 8:**
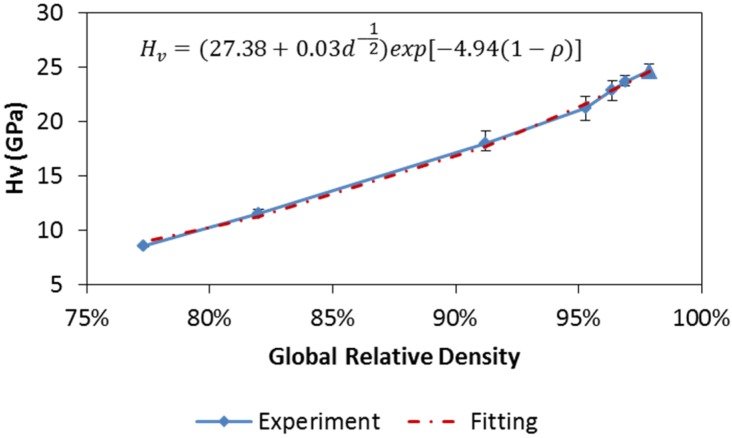
Microhardness test results with respect to relative density. Fitting equation with optimized coefficients is presented too.

Microhardness test indicates the specimen’s mechanical properties at the micro-scale. The average Vickers hardness values at three measured points (center, middle, and edge) of each tested specimen are also presented in Table 4 and correlated with the relative densities in Figure 8. The results from the double die SPS are also included as a reference. Maximum hardness is found to be 24 GPa for the specimens prepared at the maximum temperature of 1800 °C using the double die setup. The microhardness decreases as the relative density goes down. Although single-die SPS at the maximum temperature of 2100 °C gives a slightly lower hardness value of 23 GPa, this value is still higher than the results from another study conducted at the maximum temperature of 2100 °C [15].

Analogous to the analysis of the transverse rupture strength, the co-effect of the average grain size and relative density on the evolution of microhardness needs to be correctly elucidated. Literature review suggests that, the Hall-Petch relation was used to approximate the grain size from microhardness of fully dense specimens prepared at different temperatures [50]. Also, porosity was related to microhardness of ceramics sintered at the same temperature by assuming an exponential equation [51,52]. Two separate aspects are then combined in this study to form a possible representation of microhardness (GPa), *H*v, dependence on both the average grain size (µm), *d*, and the relative density, ρ, as shown below,

(14) $$H_{V}=(k_{4}+k_{5}d^{-1/2})exp\left[-b\left(1-\rho \right)\right]$$

where $k_{4},\;k_{5},\;$and b are coefficients used to fit the experimental data. The optimization of these coefficients’ values is conducted in the same way as in the rupture strength case. A parameterized Equation (14) is then obtained, as presented in Figure 8, which provides a good agreement with the experimental results. The relative density seems to be a dominant factor for the microhardness evolution (not linear, but exponential) as the obtained coefficient of the grain size ($k_{5}=0.03$) is insignificant for this specific material.

Taylor series expansion can be used to explain why the dependence of the microhardness on the relative density is exponential but appears to be linear. The exponential part of the obtained equation (see Figure 8) can be expanded at ρ=1, as follows,

(15) $$\exp\left[-4.94\left(1-\rho \right)\right]=1+4.94\left(\rho -1\right)+12.20{\left(\rho -1\right)}^{2}+O\left({\left(\rho -1\right)}^{3}\right)]$$

where the term (ρ−1) ranges from −0.22 to 0 according to Table 2, which makes high order terms (power > 1) on the right-hand side almost negligible. Therefore, the microhardness—relative density plot seems to be nearly linear but actually indicates an exponential relation.

## 5. Conclusions

The densification of a commercial zirconium carbide powder was successfully performed using single- and double-die SPS setups. High density specimens were obtained from the both setups. Both temperature and pressure effects on the densification kinetics have been elucidated. Due to the localized high temperature and applied high pressure, the double die SPS rendered a high final density but also showed the largest grain growth. The manipulation of temperature as well as pressure regimes to control the densification and the grain growth is necessary to further improve the processing outcomes.

By applying the boundary conditions of the SPS processing, an analytical densification equation that describes porosity evolution with processing time under SPS conditions was obtained based on the constitutive equation of sintering. The numerical solution of this equation is compared to the obtained experimental data in the inverse regression process in order to evaluate the power-law creep coefficients of the utilized ZrC powder. The densification mechanism is then proven to be most likely the grain boundary sliding associated with the dislocation glide controlled creep.

Transverse rupture strength of the processed ZrC specimens was tested using a recently developed technique. Its reliability has been verified by comparing to the results from other conducted studies. The rupture strength is described as a co-effect of the average grain size and relative density. These two factors are also included in the evaluation of the specimens’ microhardness.

Future works should include the densification mechanism refinement with respect to different sintering stages and the optimization of the SPS tooling setups in order to eliminate residual porosity and achieve better mechanical properties of the processed specimens. The optimized setup can be applied also to improve the sintering efficiency of other refractory ceramics. A combination of the macroscopic phenomenological approach and a high resolution microscopic analysis of the atomic-scale mass transport is an important direction of future research.
